# Comparative evaluation of isolation protocols for *Aloe vera*-derived extracellular vesicles

**DOI:** 10.3389/fphar.2026.1860135

**Published:** 2026-06-08

**Authors:** Jiahua Zhou, Weichao Hao, Zhuoya Zhang, Hao Zhang, Yi Kong, Xicheng Wang, Zhe Wang

**Affiliations:** 1 Department of Oncology, The First Affiliated Hospital of Guangdong Pharmaceutical University, Guangzhou, China; 2 Faculty of Pharmaceutical Sciences, Shenzhen University of Advanced Technology (SUAT), Shenzhen, China

**Keywords:** *Aloe vera*, cellulase, extracellular vesicles, isolation, method optimization

## Abstract

The primary objective of this investigation was to systematically evaluate four distinct protocols for extracting extracellular vesicles (EVs) from *Aloe vera*, aiming to establish a methodology that maximizes both yield and purity. Fresh *Aloe vera* gel served as the source material for EV isolation via four approaches: (A) Conventional ultracentrifugation (UC); (B) UC preceded by cellulase digestion; (C) a hybrid approach involving cellulase treatment, membrane filtration, and subsequent UC; and (D) cellulase pretreatment integrated with the EXODUS technique. Characterization of the harvested vesicles was performed using BCA protein quantification, transmission electron microscopy (TEM), and nanoflow cytometry. Morphological analysis confirmed that EVs obtained through all four strategies possessed intact lipid bilayers with diameters ranging from 50 to 100 nm. However, protocol (A) resulted in significantly lower particle recovery and exhibited an elevated protein-to-particle ratio relative to protocols (B) (C), and (D). Statistical analysis revealed no substantial variations in particle concentration or purity among methods (B) (C), and (D). Collectively, these findings indicate that enzymatic degradation of the *Aloe vera* matrix using cellulase prior to isolation markedly enhances EV recovery and purity. Compared to direct ultracentrifugation, cellulase-assisted methods yield higher particle counts, minimize co-isolated contaminants, and produce a more homogeneous size distribution.

## 1 Introduction

Extracellular vesicles (EVs) represent a class of nanoscale, lipid-bilayer-enclosed structures secreted by a wide array of cellular sources, including plant tissues. These vesicles function as critical mediators in intercellular signaling, immune modulation, and cross-kingdom biological interactions (Yanez-Mo et al., 2015). Among plant-derived EVs, those extracted from *Aloe vera* have attracted considerable research attention owing to their robust therapeutic potential, which includes anti-inflammatory, antioxidant, and tissue-regenerative capabilities (Ghosh et al., 2026; Ramirez et al., 2024), positioning them as novel tools in the evolving landscape of biocompatible drug carriers-a theme recently highlighted in comparative studies between plant and mammalian EVs (Maricchiolo et al., 2026).

Obtaining EVs of high purity is an essential prerequisite for ensuring the reliability of downstream functional assays and characterization studies. Traditional isolation strategies encompass ultracentrifugation, polymer-based precipitation, and size-exclusion ultrafiltration, while novel technologies such as the ultrafast EXODUS system have recently emerged (Chen et al., 2021). Nevertheless, isolating EVs from *Aloe vera* gel presents unique challenges due to its intricate, polysaccharide-dense matrix, which often hinders efficient vesicle release and purification (Kim and Park, 2022). To address this, enzymatic pretreatment-specifically using cellulase-has been proposed to degrade rigid plant cell wall components, thereby facilitating the liberation of encapsulated vesicles (Zhang et al., 2016).

In the current study, we performed a comprehensive comparative analysis of four isolation methodologies for *Aloe vera*-derived EVs: (i) conventional ultracentrifugation (UC); (ii) cellulase-assisted ultracentrifugation (CA-UC); (iii) cellulase-assisted filtration coupled with ultracentrifugation (CAF-UC); and (iv) cellulase-assisted EXODUS (CA-EXODUS). The efficacy of each protocol was rigorously assessed based on key metrics, including nanoparticle yield, extent of protein contamination, and preservation of vesicular morphological integrity.

## 2 Materials and methods

### 2.1 Sample preparation

Fresh *Aloe vera* leaves (American Curacao variety, 7-year-old plants, each leaf approximately 300 g; total of ∼2.5 kg per batch) were meticulously washed with ultrapure water. The leaves were peeled, and the gel was cut into small pieces. Mechanical juicing was performed using a commercial juicer (Midea, model MJ-WBL2521H, China) equipped. The expressed crude juice (1,000 mL) was subsequently allowed to sediment at 4 °C for 12 h to facilitate the removal of coarse debris. The resulting supernatant served as the starting material for subsequent extracellular vesicle (EV) isolation.

### 2.2 EVs isolation methods

#### 2.2.1 Method A: conventional ultracentrifugation (UC)

To eliminate macromolecular debris, the supernatant underwent sequential differential centrifugation at 4 °C: 1,000×g for 10 min, 3,000×g for 20 min, 5,000×g for 30 min, and finally 10,000×g for 40 min. Approximately 120 mL of the clarified supernatant was transferred into polycarbonate ultracentrifuge tubes and spun at 100,000×g for 70 min at 4 °C using a fixed-angle rotor (Beckman Coulter, Type 45 Ti). The resultant pellet was gently resuspended in 1×PBS (1 mL per pellet) to yield roughly 2 mL of EV suspension, which was then aliquoted and preserved at −80 °C for future analyses.

#### 2.2.2 Method B: cellulase pretreatment + UC

An aliquot of the sedimented juice (1,000 mL) was incubated with cellulase (Solarbio, China, catalog No. C8270) at a final concentration of 1 mg/mL (20 mL of enzyme solution per 1,000 mL juice). The mixture was incubated at 37 °C for 30 min to digest residual plant cell wall constituents. Subsequently, the sample proceeded through identical differential and ultracentrifugation steps as described in Method A (Section 2.2.1) to recover the EV fraction.

#### 2.2.3 Method C: cellulase pretreatment + filtration + UC

After the same cellulase treatment as in Method B, the supernatant was further clarified by filtration through a 0.22 μm size membrane to eliminate remaining large particulates and aggregates. Ultracentrifugation was then performed exactly as described in Section 2.2.1 to isolate EVs. The resulting EV pellet was resuspended in 1 mL of 1×PBS per pellet (two pellets combined into 2 mL) and stored at −80 °C.

#### 2.2.4 Method D: cellulase pretreatment + EXODUS

To achieve high-purity EV isolation, 50 mL of cellulase-treated (prepared as in Method B) and pre-centrifuged aloe juice was loaded into the EXODUS system (model H600, Shenzhen Huixin Lifetechnologies Co., Ltd. China) (Chen et al., 2021). The following parameters were applied: program Feeble- SA. A03, M-chip. This device utilizes a double-coupled oscillatory membrane mechanism to effectively separate EVs from soluble contaminants. Upon completion of the automated isolation cycle, the concentrated EV preparation was washed with 1×PBS under consistent operational parameters. Approximately 400 μL of purified EVs was collected and maintained at −80 °C for downstream characterization and functional assays.

### 2.3 EVs characterization

#### 2.3.1 Transmission electron microscopy (TEM)

The morphological features of isolated *Aloe vera* EVs were assessed via transmission electron microscopy (Hitachi HT7800, Japan). Briefly, 10 μL of EV suspension was applied onto a carbon-coated copper grid and allowed to adsorb for 1 min. Excess fluid was carefully wicked away using filter paper. The specimen was then negatively stained with 10 μL of 2% uranyl acetate for 1 min, after which residual stain was blotted off. Grids were air-dried at ambient temperature for several minutes prior to imaging at an accelerating voltage of 80 kV.

#### 2.3.2 Nanoparticle size and concentration analysis (nanoflow cytometry)

Particle size profiles and concentrations of *Aloe vera* EVs were quantified using nanoflow cytometry (NanoFCM, Xiamen, China). EV samples were appropriately diluted in sterile PBS to fall within the instrument’s optimal detection window. Before analysis, the nanoflow cytometer was calibrated and validated using standard 100 nm silica nanoparticles. To avoid needle clogging, serial dilutions of EV samples were evaluated. Particle concentration data and size distribution histograms were generated and interpreted using the dedicated NFCM software suite.

#### 2.3.3 Protein extraction and quantification

To evaluate the yield and purity of the isolated extracellular vesicle (EV) fractions, total protein content was measured. EV suspensions were quickly thawed at 37 °C and combined with five volumes of RIPA lysis buffer containing protease inhibitors. Following vortexing, samples were incubated on ice for 30 min with periodic mixing to guarantee complete cell lysis. Protein levels were assessed using a bicinchoninic acid (BCA) assay kit (Thermo Fisher Scientific, USA), strictly adhering to the supplier’s protocol. A standard curve was generated employing bovine serum albumin (BSA) standards. Specifically, 20 μL of either diluted sample or standard was dispensed into microplate wells, followed by the addition of 200 μL BCA working solution. After a 30-min incubation at 37 °C, absorbance readings were obtained at 562 nm utilizing a microplate reader (BioTek, USA). Sample protein concentrations were subsequently derived from the established standard curve.

#### 2.3.4 Coomassie blue-stained SDS-PAGE

To compare protein contaminants in *Aloe vera*-derived extracellular vesicles (EVs) from four isolation methods, we used SDS-PAGE with Coomassie staining. EV aliquots (30 μL, 50 μg) and crude Aloe juice were denatured in Laemmli buffer with β-mercaptoethanol at 95 °C for 10 min, then run on a 10% resolving gel with a 5% stacking gel at 80 V for 30min, then 120 V for 60 min using a Mini-PROTEAN Tetra system (Bio-Rad, USA). Gels were stained in 0.1% Coomassie R-250 for 60 min, destained, and imaged the gel.

### 2.4 Statistical analysis

Results are expressed as mean ± standard error of the mean (SEM). Comparisons between two groups utilized Student’s t-test, whereas multiple group comparisons employed analysis of variance (ANOVA). Statistical significance was defined as *p* < 0.05, with asterisks denoting specific thresholds: *p < 0.05, **p < 0.01, and ***p < 0.001. All experimental procedures were performed in triplicate.

## 3 Results

### 3.1 Evaluation of isolation efficiency: particle yield and purity

**FIGURE 1:**
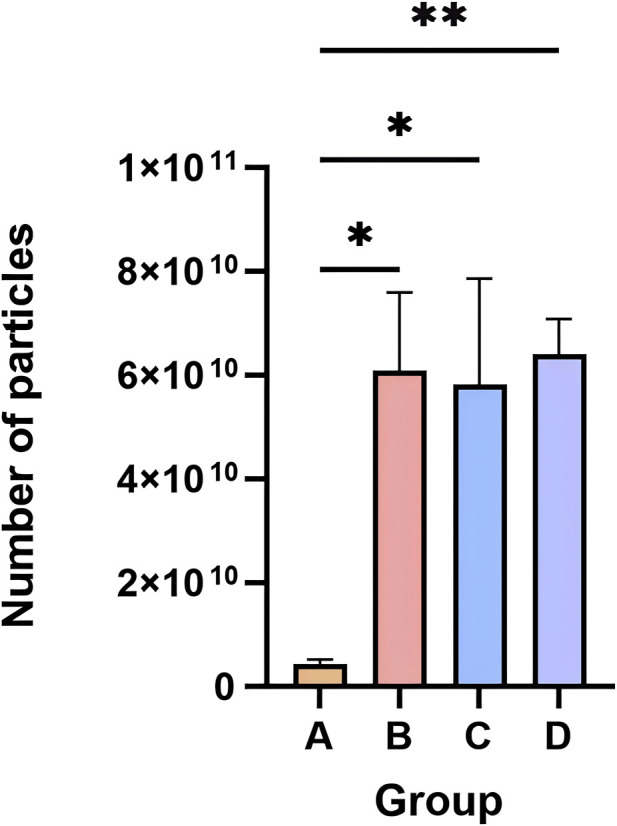
Comparison of particle yield of *Aloe vera* EVs isolated by four different methods. The concentration of extracellular vesicles (EVs) isolated from *Aloe vera* gel was quantified by nanoflow cytometry. Methods are: (A) Conventional Ultracentrifugation (UC), (B) Cellulase + UC, (C) Cellulase + Filtration + UC, and (D) Cellulase + EXODUS. Data are presented as mean ± SEM (n ≥ 3). *p < 0.05, **p < 0.01.

We quantitatively assessed four distinct isolation protocols by examining particle yield and purity, metrics defined by nanoparticle concentration and the particle-to-protein ratio (Thery et al., 2018), respectively. Nanoflow cytometry analysis uncovered substantial variations in the recovery of *Aloe vera*-derived EVs (Figure 1). Method A (Conventional ultracentrifugation, UC) produced the minimal particle count, approximating 2 × 10^10^. Conversely, protocols involving cellulase pre-treatment (Methods B, C, and D) achieved significantly elevated and comparable particle concentrations, spanning from 8 × 10^10^ to 1 × 10^11^. Importantly, statistical analysis revealed no significant divergence in particle yield among Methods B, C, and D.

Coomassie-stained SDS-PAGE (Supplementary Figure S1) showed complex protein profiles in *Aloe vera* juice controls, while all EV preparations had simpler patterns. Method A had bands at 55–72, 34–43, and 26 kDa, reflecting a relatively higher level of isolated contaminants. Method B and C showed highly similar protein patterns, with major bands at 34–43 kDa region. Method D had weaker 34–43 kDa bands, suggesting EXODUS removes those proteins.

**FIGURE 2:**
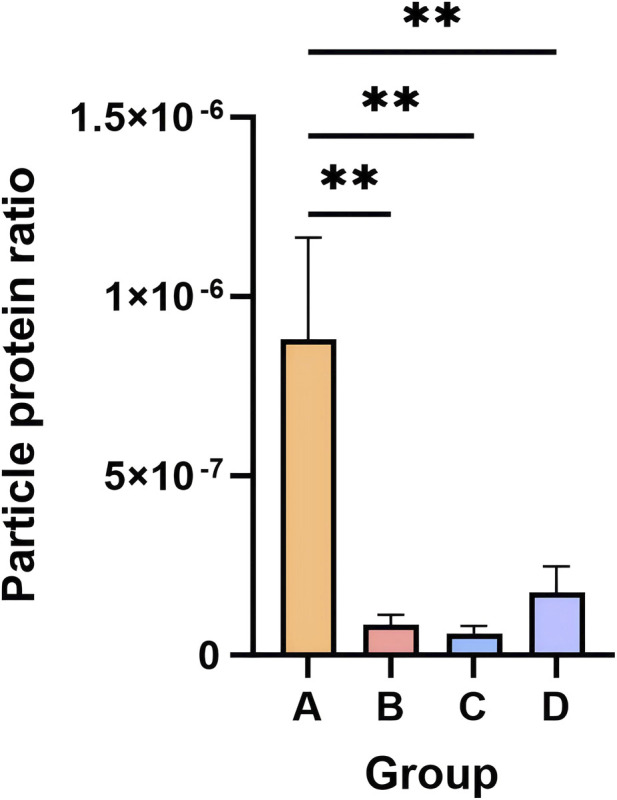
Purity assessment of isolated *Aloe vera* EV preparations. The purity of EV isolates was evaluated by the particle-to-protein ratio, calculated from the particle concentration (nanoflow cytometry) and total protein concentration (BCA assay). A higher ratio indicates a purer EV preparation with less co-isolated soluble protein contamination. Methods are: (A) Conventional Ultracentrifugation (UC), (B) Cellulase + UC, (C) Cellulase + Filtration + UC, and (D) Cellulase + EXODUS. Data are presented as mean ± SEM (n ≥ 3). *p < 0.05, **p < 0.01.

Regarding purity, calculated via the particle-to-protein ratio (Figure 2), findings mirrored the yield data. Method A generated EV preparations with the lowest purity, characterized by a markedly reduced particle-to-protein ratio. In contrast, Methods B, C, and D demonstrated ratios approximately three times higher than Method A, suggesting a considerable decrease in co-isolated soluble protein contaminants. Consistent with yield observations, purity levels remained comparable across the three cellulase-mediated approaches.

### 3.2 Morphological and size characterization

**FIGURE 3:**
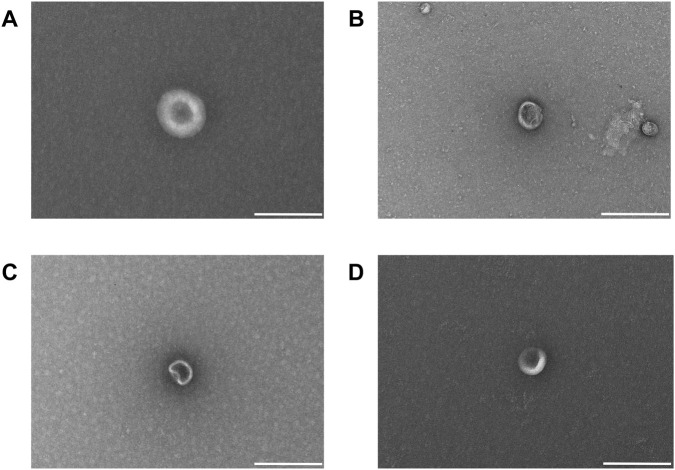
Morphology of EVs observed by TEM Representative transmission electron microscopy (TEM) images of *Aloe vera*-derived extracellular vesicles (EVs) isolated by four different protocols. **(A)** Conventional Ultracentrifugation (UC), **(B)** Cellulase + UC, **(C)** Cellulase + Filtration + UC, and **(D)** Cellulase + EXODUS. Scale bars = 200 nm.

The morphological integrity of *Aloe vera*-derived EVs from four protocols was assessed via TEM. All methods yielded vesicles with visible lipid bilayers and typical cup-shaped or spherical morphology (Figure 3). However, differences in integrity and background cleanliness were observed. Method A produced EVs with clear bilayers, but some vesicles collapsed or aggregated, and backgrounds contained non-vesicular debris. Methods B and C yielded well-preserved EVs with cleaner backgrounds due to cellulase pretreatment. Method D showed typical morphology and the cleanest background.

**FIGURE 4:**
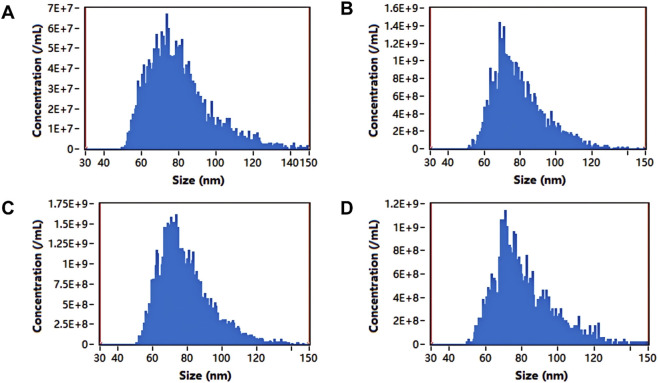
Particle size distribution of EVs measured by NTA All four methods yielded EVs with a primary size range of 50–100 nm. **(A)** Conventional Ultracentrifugation (UC), Mean: 80.2 ± 16.4 nm, **(B)** Cellulase + UC, Mean: 79.9 ± 14.2 nm, **(C)** Cellulase + Filtration + UC, Mean: 78.9 ± 14.8 nm, and **(D)** Cellulase + EXODUS, Mean: 81.2 ± 16.2 nm.

Particle size distribution and concentration were analyzed by nanoparticle tracking analysis (NTA) (Figure 4). All four methods yielded EV populations primarily sized 50–100 nm, aligning with typical small EVs. However, subtle distribution differences were observed. Method A showed a broader distribution, suggesting larger aggregates. Method B, C and D had a narrower distribution centered around 70–80 nm.

## 4 Discussion and conclusion

This study evaluated four distinct protocols for isolating extracellular vesicles (EVs) from the complex matrix of *Aloe vera* gel. Our findings conclusively demonstrate that enzymatic pre-digestion using cellulase is critical for maximizing both the recovery yield and purity of *Aloe vera*-derived EVs (Zhang et al., 2016). The suboptimal particle retrieval and reduced purity associated with Method A (conventional ultracentrifugation, UC) are likely attributable to the dense, polysaccharide-rich architecture characteristic of *Aloe vera* gel. This structural network appears to sequester a substantial proportion of EVs, impeding efficient sedimentation while concurrently co-precipitating with vesicles, thereby introducing significant proteinaceous contamination.

The marked improvements in particle concentration and purity observed with Methods B, C, and D highlight the effectiveness of cellulase in hydrolyzing cellulose-based cell wall constituents, thereby liberating trapped vesicles and reducing soluble impurities (Liu et al., 2020). In terms of viscosity, this enzymatic degradation facilitates superior separation of EVs from co-isolated soluble proteins during subsequent ultracentrifugation or purification steps.

The comparable performance of Methods B, C, and D regarding both yield and purity represents a key finding. This suggests that once the *Aloe vera* matrix is disrupted by cellulase, the core principle of size-based fractionation whether implemented via Method B (Cellulase + UC), membrane filtration combined with UC (Method C), or the sophisticated oscillatory filtration employed by EXODUS (Method D)-is equally effective in recovering a uniform EV population. Therefore, for routine laboratory-scale isolation of *Aloe vera* EVs, the simple and accessible Method B (Cellulase + UC) emerges as the most practical and cost-effective approach. While EXODUS (Method D) offers a robust automated platform, its advantages in throughput and potential for ultra-high purity are primarily relevant for large-scale production or applications requiring exceptional purity, rather than standard research workflows. Attaining high purity in cellulase-treated samples is essential for downstream functional assays and applications, as residual proteins may obscure intrinsic EV biological activities or induce non-specific responses.

In conclusion, the effective isolation of extracellular vesicles (EVs) from *Aloe vera* is fundamentally dependent on the selected extraction strategy. Traditional ultracentrifugation (Method A) demonstrates significant limitations in recovering high-purity EVs from this plant matrix. The integration of a cellulase pre-digestion step represents a straightforward yet pivotal modification; by degrading the gelatinous structural network, this approach markedly enhances both the yield and purity of the isolated vesicles. Among the evaluated protocols, Method B (Cellulase combined with Ultracentrifugation) offers an optimal balance of high recovery efficiency, superior purity, and practical feasibility, making it well-suited for standard laboratory settings. Consequently, we propose that cellulase-assisted methodologies be adopted as the benchmark for the isolation of *Aloe vera*-derived extracellular vesicles.

## Data Availability

The original contributions presented in the study are included in the article/Supplementary Material, further inquiries can be directed to the corresponding authors.
