# Supplementary material for: Comparative evaluation of isolation protocols for Aloe vera-derived extracellular vesicles
Source: Front Pharmacol. 2026 Jun 8;17:1860135. doi: 10.3389/fphar.2026.1860135 (PMC13284160; doi:10.3389/fphar.2026.1860135)
Supplement: Supplementary file 1 [file Image1.pdf]

Supplementary data for

Comparative Evaluation of Isolation Protocols for Aloe vera-Derived Extracellular Vesicles

Jiahua Zhou[1†], Weichao Hao[1†], Zhuoya Zhang[1], Hao Zhang[1], Yi Kong[2*], Xicheng Wang[1*], Zhe Wang[1*]

1.Department of Oncology, the First Affiliated Hospital of Guangdong Pharmaceutical University, Guangzhou 510000, China

2.Faculty of Pharmaceutical Sciences, Shenzhen University of Advanced Technology (SUAT), Shenzhen 518107, China

[†] **These authors contributed equally to this work.**

*** Correspondence:**

Zhe Wang: wangzhe0409@gmail.com, wangzhe@gdpu.edu.cn

Xicheng Wang: **13902400598@126.com**

Yi Kong: kongyi@suat-sz.edu.cn

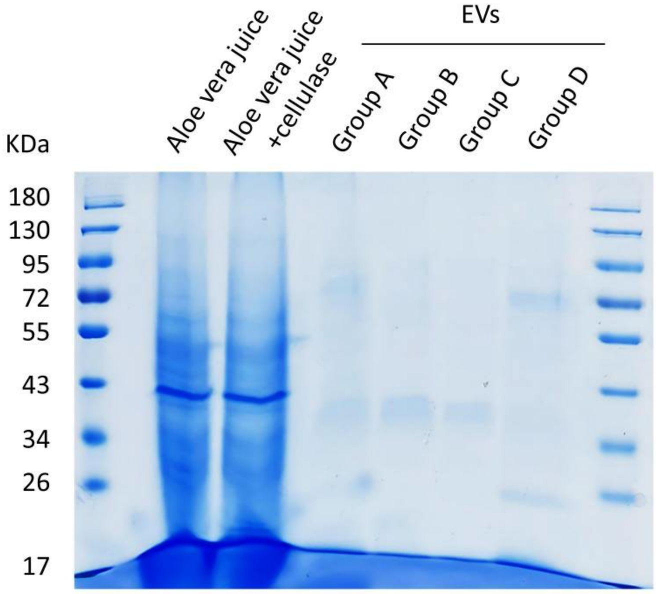

Figure S1. Coomassie Blue-stained SDS-PAGE gel comparing protein profiles of Aloe vera crude juice and EVs isolated by four different protocols.

The crude juice samples show broad, complex protein bands across the entire molecular weight range (17-180 kDa), with slightly weaker intensity after cellulase treatment. EV preparations exhibit substantially simplified protein profiles. Group A shows major bands at 55-72, 34-43, and 26 kDa. Group B and C display similar patterns predominantly at 34-43 kDa. Group D shows major bands at 55-72 and 26 kDa, with markedly weaker bands at 34-43 kDa. Group A: Conventional Ultracentrifugation (UC), Group B: Cellulase + UC, Group C: Cellulase + Filtration + UC, and Group D: Cellulase + EXODUS.
